# Isolated panniculitis with vasculitis of the male breast suspicious for malignancy on CT and ultrasound: a case report and literature review

**DOI:** 10.1186/2193-1801-3-642

**Published:** 2014-10-30

**Authors:** Wei-Hsin Yuan, Anna Fen-Yau Li, Hui-Chen Hsu, Yi-Hong Chou

**Affiliations:** Division of Radiology, Taipei Municipal Gan-Dau Hospital (Managed by Taipei Veterans General Hospital), Taipei, Taiwan; Department of Radiology, Taipei Veterans General Hospital, No. 201, Sec. 2, ShiPai Road, 11217 Taipei City, Beitou District Taiwan; Department of Pathology, Taipei Veterans General Hospital, Taipei, Taiwan; Division of Medical Imaging for Health Management, Cheng Hsin General Hospital, Taipei, Taiwan; School of Medicine, National Yang Ming University, Taipei, Taiwan

**Keywords:** Panniculitis with vasculitis, Male breast cancer, Sonography, Computed Tomography (CT)

## Abstract

**Introduction:**

We report a case of a 54-year-old male patient with a hard, painful nodule within his right breast which was misdiagnosed preoperatively as breast cancer.

**Case description:**

Preoperative work-up included physical examination, non-contrast chest computed tomography (CT), sonography, and sono-guided breast biopsy. Isolated breast panniculitis with vasculitis (BPWV), a rare disease, was diagnosed by histopathologic examination of tissue obtained from excisional biopsy.

**Discussion and Evaluation:**

Subcutaneous panniculitis with or without vasculitis, a condition of nonsuppurative inflammatory process involving the subcutaneous fat layer of skin, is related to different causes. A palpable benign male breast lesion resembling a malignancy includes gynecomastia, panniculitis with or without vasculitis, fat necrosis, ruptured epidermal cyst, pseudoangiomatous stromal hyperplasia, subareolar abscess, intraductal papilloma, hematoma, and atypical fibroadenoma. To make an accurate preoperative diagnosis of a male breast mass, a physician has to carefully analyze various imaging findings. The cases of BPWV may present as an isolated breast lesion or as a component of a systemic disease. The diagnosis of the reported patient was compatible with an isolated BPWV because panniculitis and/or vasculitis were not present at other sites or organs at the time of diagnosis or during follow-up.

**Conclusions:**

Excisional biopsy and clinical data can provide the correct diagnosis and determined the appropriate treatment strategy of a male BPWV.

## Background

Greater than 99% of breast lesions encountered in men are benign (Yitta et al. 2010). The most common benign lesion of the male breast is gynecomastia located within the subareolar region of the breast (Ng et al. 2014). Once a nodule or mass develops within the other quadrants of the male breast, the most critical diagnosis is breast cancer, however, malignant lesions of the male breast are uncommon (Ng et al. 2014).

Mammography, ultrasonography, and magnetic resonance imaging (MRI) are the most common modalities used in the work-up of a breast mass. Ultrasonography is widely used in the evaluation of a breast lesion since it is convenient, noninvasive, inexpensive, and does not involve the use of ionizing radiation. However, clinicians and radiologists need sufficient imaging experience to correctly interpret the findings associated with a breast lesion using this modality. Chest CT is not routinely used for evaluation of a male breast mass. With increasing use of chest CT for the diagnosis of thoracic lesions, however, breast incidentalomas are more frequently encountered (Bach et al. 2013).

This report emphasized the proper diagnostic work-up of a solitary nodule within a male breast including imaging findings from sonography, chest CT, mammography, and breast magnetic resonance imaging (MRI). In addition, the clinical pattern, pathogenesis, treatment, and prognosis of isolated breast panniculitis with vasculitis were reviewed.

## Case description

A 54-year-old man, with a history of type 2 diabetes mellitus, poorly controlled chronic hypertension, and chronic kidney disease on hemodialysis, was evaluated for a one-month history of a painful, non-mobile, subcutaneous nodule within the inferior and medial aspect of his right breast. The patient also complained of general pruritus but no malaise, joint pain or muscle pain. Body temperature, respiration, and pulse were normal. His blood pressure was elevated at 182/94 mm Hg. His body height, body weight, and body mass index were 165 cm, 52 kg, and 19.1, respectively.

On physical examination, a firm, superficial, erythematous nodule measuring approximately 1 × 2 cm was noted within the lower, inner quadrant of the right breast. There was no nipple discharge, history of trauma, or cancer history. Given the possibility of breast carcinoma, the patient was admitted for further evaluation. Upon admission, initial laboratory results revealed: BUN, 40 mg/dl; creatinine, 28.63 mg/dl, C-reactive protein, 7.04 mg/L; hemoglobin, 6.3 g/dl; and a white blood count, 6700 /ml, with a left shift.

**Figure 1:**
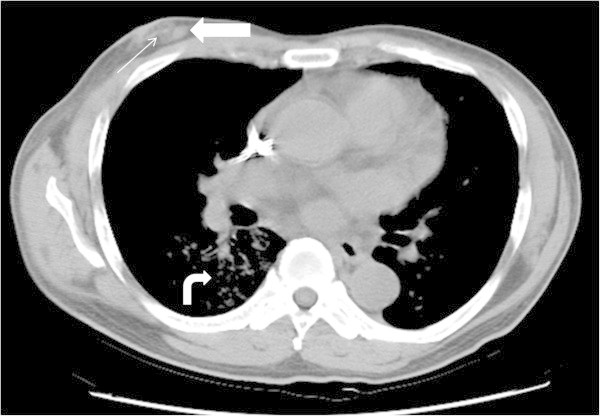
**Breast panniculitis with vasculitis on axial non-contrast CT scan of chest.** An oval nodule is located within the central inner region of the right breast (thick arrow) with increased infiltration of the subcutaneous adipose tissue (thin arrow). Pulmonary alveolar infiltrations within the right lower lobe of lung are also present (curved arrow).

Chest X-ray upon admission showed increased patchy infiltrates bilaterally and a non-contrast chest CT was performed for further evaluation. The chest CT revealed diffuse patchy airspace consolidation and patchy pulmonary alveolar infiltrates with fine interstitial thickening over both lung fields, most severe within the left upper lobe and right lower lobe (Figure 1). The primary diagnosis was atypical pneumonia. Differential diagnosis included asymmetric pulmonary edema and multifocal bronchoalveolar carcinoma.

Sputum culture, sputum cytology, serum mycoplasma antibody, and legionella antibody were performed. Azithromycin and augmentin were begun for possible atypical pneumonia. Sputum cytology was negative for malignant cells. Sputum culture, however, revealed Klebsiella pneumoniae and Klebsiella oxytoca (without tubercle bacillus). Appropriate antibiotic therapy was continued and repeat serial chest X-rays, performed during his hospital stay, showed resolution of the alveolar infiltrates, bilaterally.

**Figure 2:**
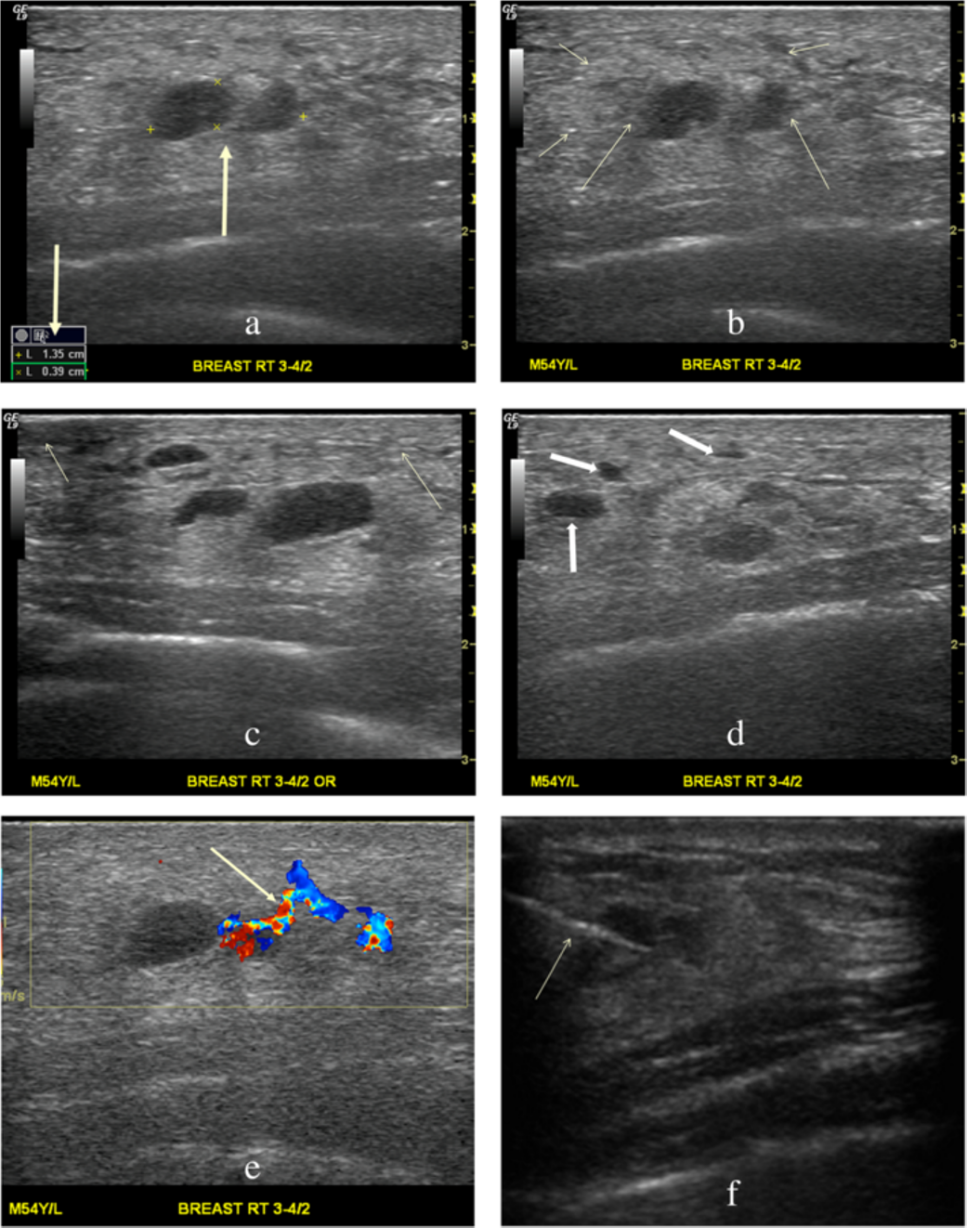
**Breast panniculitis with vasculitis on ultrasound. a**. A heterogeneously hypoechoic nodule within the right breast measures 1.35 cm × 0.39 cm in size (arrows). **b**. A faint echogenic interface (short arrows) and partially instinct margin (long arrows) of the nodule are present. **c**. Normal skin (short arrow) and skin thickening (long arrow) over the nodule are observed. **d**. Additional hypoechoic satellite nodules (short arrows) are visualized. **e**. Blood flow signals increase in and around the nodule on color Doppler (arrow). **f.** A sono-guided biopsy of the nodule with an 18-gauge core biopsy needle (arrow) is performed.

**Figure 3:**
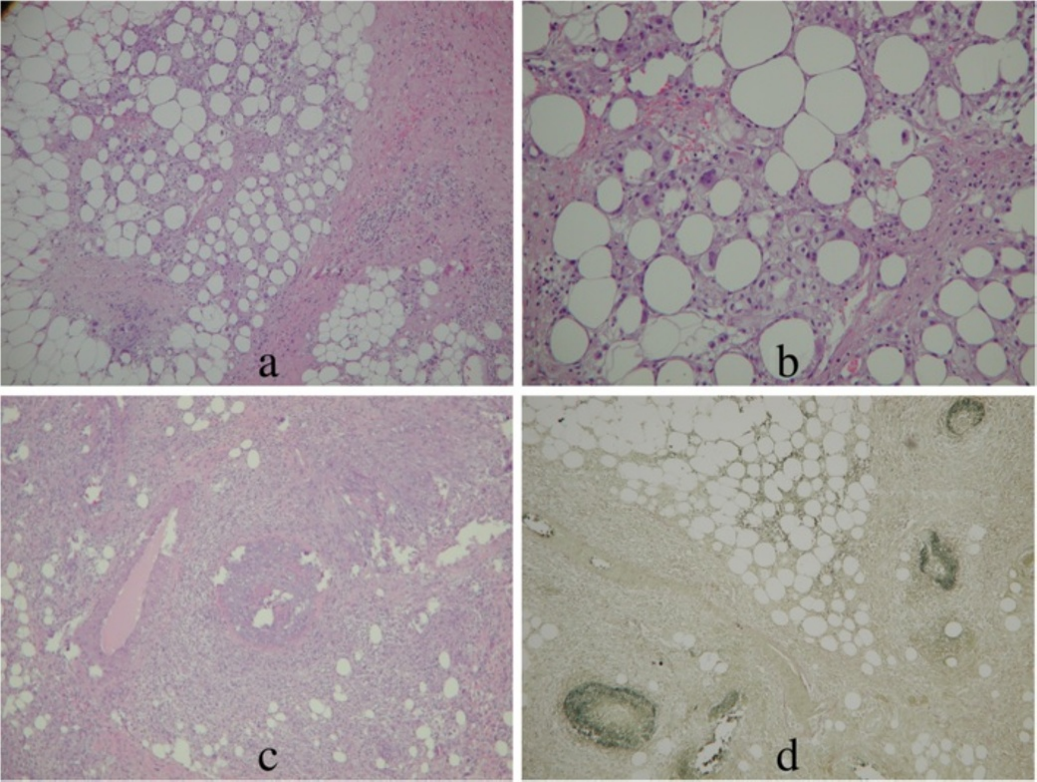
**Pathologic specimens of breast panniculitis with vasculitis from the excisional biopsy. a**. and **b**. Histologic section shows primarily fibroadipose tissue within the right breast with chronic inflammatory cells infiltration and fibrotic change. (hematoxylin & eosin, original magnification, 40× and 100×). **c**. Chronic inflammatory cells infiltrate around and in a small vessel with the lumen obliterated (hematoxylin & eosin, original magnification, 100×). **d**. A vasculitis involves a medium size artery and small vessels (Elastic van Gieson stain, original magnification, 100×).

Incidentally noted on chest CT was a small oval nodular lesion measuring approximately 1.0 cm × 0.6 cm over the inferior and medial aspect of the right breast with infiltration within the subcutaneous adipose tissue (Figure 1). As the nature of the breast nodule was unclear, a breast ultrasound (GE Healthcare, Milwaukee, WI) was recommended for further evaluation. Breast sonography revealed an oval, heterogeneous, predominantly hypoechoic nodule within the right breast measuring 1.35 cm × 0.39 cm in size between 3 and 4 o’clock, 2 cm from the nipple, with a faint echogenic interface, partially indistinct margin, and increased blood flow signals on color Doppler (Figure 2). Skin thickening and additional hypoechoic satellite nodules were noted in the surrounding area (Figure 2). As this presentation was felt to be unusual in a male breast, breast malignancy was suspected. As the patient refused mammography or MRI, a sono-guided biopsy of the lesion was performed using an 18-gauge core biopsy needle (Figure 2). Pathology from four strips tissue revealed fibroadipose tissue and hemorrhage with inflammatory cell infiltration. As the pathology was considered indeterminant, an excisional biopsy of the mass was performed. Pathology from the excisional biopsy confirmed breast panniculitis, consisting of fibroadipose tissue with prominent inflammation and fibrotic change in a vague perivascular pattern (Figure 3). Hemorrhage and hemosiderin deposition were also present. Histologic section further revealed a vasculitis involving medium size arteries and small vessels without giant cells or granuloma formation (Figure 3).

Mycoplasma antibody titer, legionella antibody titer, anti-neutrophil cytoplasmic antibody (ANCA), autoimmune markers, carcinoembryonic antigen, cancer antigen 15–3, prostatic specific antigen, immunoglobulin G, immunoglobulin A, human immunodeficiency virus antibody titer, hepatitis C antibody titer, and hepatitis B surface antigen titer were all negative. A non-contrast head CT was also negative, performed as part of a headache work-up. Renal sonogram revealed increased cortical echogenicity of both kidneys compatible with chronic renal parenchymal disease. No definite renal infarctions were identified.

The lesion within the right breast resolved after excisional biopsy without further treatment. As the diagnosis was compatible with an isolated breast panniculitis with vasculitis (BPWV), the patient did not receive systemic corticosteroids or anti-inflammatory therapy during admission or after discharge and his breast symptoms did not recur during the subsequent 5 years of clinical follow-up.

## Discussion and Evaluation

Subcutaneous panniculitis refers to a broad spectrum of nonsuppurative inflammatory processes involving the subcutaneous fat layer of the skin (Ho and Lam 2002; Walsh et al. 2008; Pinho et al. 2007). Normal subcutaneous fat is incorporated as fat cells arranged into lobules surrounded by fibrous septa containing small blood vessels, lymphatic channels, and nerves (Fernando et al. 2003).

Clinically, subcutaneous panniculitis usually presents as tender or painless nodules within an extremity, trunk, neck, face, or breast (Ho and Lam 2002; Walsh et al. 2008; Fernando et al. 2003). Subcutaneous panniculitis typically is associated with the fat of the lower extremities (Pinho et al. 2007). However, subcutaneous panniculitis in the breast is rare and a palpable breast abnormality can be misjudged as cancer at clinical examination (Cho and Park 2013).

Subcutaneous panniculitis is divided into five histopathologic patterns (Ho and Lam 2002; Walsh et al. 2008; Fernando et al. 2003; Ter Poorten and Thiers 2002; Ferrara et al. 2013): 1) lobular panniculitis without vasculitis, 2) lobular panniculitis with vasculitis, 3) septal panniculitis without vasculitis, 4) septal panniculitis with vasculitis, and 5) mixed panniculitis involving septa and lobules. Lobular panniculitis may be related to idiopathic Weber-Christian disease, trauma, intragenic injections, cold exposure, sickle cell disease, alpha-1 antitrypsin deficiency, pancreatic disease, systemic lupus erythema, scleroderma, erythema induratum, and paraneoplastic disease. Septal panniculitis may be associated with erythema nodosum, eosinophilic fasciitis, and polyarteritis nodosa. Mixed panniculitis may occur in lupus profundus, and subcutaneous sarcoidosis. Once a vasculitis has been detected within the subcutaneous tissue by histopathology, polyarthritis nodosa, erythema nodosum, and nodular vasculitis should be considered (Ferrara et al. 2013; Gilchrist and Patterson 2010).

The histologic findings in our case were compatible with panniculitis with vasculitis involving medium-sized arteries and small vessels. As neither giant cells nor granuloma formation were demonstrated in our patient, erythema nodosum and nodular vasculitis were considered less likely. However, polyarteritis nodosa, microscopic polyangiitis, or other forms of vasculitis were considered.

Sonographic findings of subcutaneous panniculitis, with or without vasculitis, vary with pathologic stage and reflect the degree of fibrosis (Baillie and Mok 2004; Taboada et al. 2009; Tan et al. 2006). During the initial stage, subcutaneous fat cells are destroyed with subsequent hemorrhage and inflammatory infiltrate. During the subsequent reparative phase, fibroblasts proliferate within the surrounding tissues; during the final stage, fibrosis gradually replaces fat necrosis with little or no calcification. On sonography, subcutaneous breast panniculitis usually appears as a hyperechoic focus within the hypoechoic subcutaneous fat, leading to non-uniformity of the subcutaneous tissue during the initial stage of pathology (Ho and Lam 2002; Taboada et al. 2009). During the subsequent reparative and final stages, the margins of the lesions range from well defined, to indistinct or spiculated, depending on the degree of fibrosis. Subcutaneous fat necrosis in panniculitis may present as a complex mass with mural nodules, an isoechoic mass, a hypoechoic solid mass, or an anechoic cystic mass with posterior acoustic enhancement or shadowing (Fernando et al. 2003; Cho and Park 2013). Posterior acoustic enhancement may be associated with serous fluid within fat necrosis, and posterior acoustic shadowing may related to oil cysts with calcifications (Fernando et al. 2003; Taboada et al. 2009).

Subcutaneous masses with increased echogenicity are almost always benign (Baillie and Mok 2004). Benign male breast lesions resembling a malignancy on gray-scale and Color Doppler sonography show at least one of six criteria: an irregular shape, non-circumscribed margins, a heterogeneous internal echotexture, an echogenic interface, posterior acoustic shadowing, and increased internal vascularity (Yitta et al. 2010; Cho and Park 2013; Tan et al. 2013). Mimickers of breast malignacy include gynecomastia, fat necrosis, ruptured epidermal cyst, pseudoangiomatous stromal hyperplasia, subareolar abscess, intraductal papilloma, hematoma, and atypical fibroadenoma (Yitta et al. 2010; Ng et al. 2014; Cho and Park 2013). The breast lesion in our patient presented with four of six malignant features on sonography.

The benign signs of breast incidentalomas on standard chest CT scans include small size (mean ± standard deviation, 13.4 ± 8.7 mm), oval shape, calcifications, no axillary lymphadenopathy, and inhomogeneous contrast enhancement (Bach et al. 2013). However, there is no significant difference between benign and malignant breast lesions with regards to margin characteristics on chest CT (Bach et al. 2013). Therefore, non-contrast chest CT is insufficient to differentiate between a benign vs. malignant breast lesion in our patient. Although neither mammography nor breast MRI were performed in our case, mammography and MRI findings of subcutaneous panniculitis, with or without vasculitis, can also vary with different pathologic stage and reflect the degree of fibrosis (Pinho et al. 2007; Baillie and Mok 2004; Taboada et al. 2009; Tan et al. 2006).

Mammography is superior to sonography regarding the evaluation of microcalcifications (Pinho et al. 2007). Breast panniculitis (with or without vasculitis) on mammography has been described as a focal asymmetry, mass, or radiolucent lesion with suspicious microcalcifications (curvilinear, branching, punctate, or amorphous) paralleling the course of fat necrosis (Pinho et al. 2007). Coarse calcifications, radiolucent appearance, and local skin thickening on mammography are benign features of breast panniculitis. Benign features on breast MRI include local cutaneous involvement, high signal intensity on precontrast T1-weighted images and low signal intensity on precontrast fat-suppressed T1-weighted images suggesting fat, and continuous rim enhancement on MRI kinetic analysis instead of a wash-out curve (Pinho et al. 2007; Taboada et al. 2009; Sabate et al. 2006). During the reparative and final stage of the disease, BPWV could present with indistinct or spiculated margins and architectural distortion on sonography, mammography and MRI, which may be indistinguishable from breast malignancy (Pinho et al. 2007; Taboada et al. 2009; Sabate et al. 2006).

Histopathologic features of BPWV, including granulomatous and nongranulomatous manifestations and size of inflamed vessels, are not useful in predicting extent of disease (Hernandez-Rodriguez et al. 2008). Diagnosis of isolated or systemic subcutaneous panniculitis with vasculitis is established by a combination of clinical and histologic features, laboratory abnormalities, immunologic markers, exclusion of specific infectious agents, and even visceral angiography (Pinho et al. 2007; Hernandez-Rodriguez et al. 2014).

The pathogenesis of cutaneous vasculitis is mediated by serum immune complexes or antineutrophil cytoplasmic antibodies (Hernandez-Rodriguez et al. 2014). Half of the cases of BPWV have presented as an isolated breast lesion whereas the other half of cases presented as a component of a systemic disease (Hernandez-Rodriguez et al. 2008). Isolated cutaneous panniculitis with vasculitis can regress spontaneously or develop into late systemic disease, with the patient eventually developing malignant hypertension and subsequent end-stage renal disease (Pinho et al. 2007; Hernandez-Rodriguez et al. 2014; Chen and Carlson 2008; Selga et al. 2006). In our review of the literature, renal involvement was seen in 70% of cases of polyarteritis nodosa at diagnosis. Polyarteritis nodosa or microscopic polyangiitis can be considered a self-limiting disease with a relapse rate of between <10% and 57% (Hernandez-Rodriguez et al. 2014).

In our case, BPWV did not coincide with chronic hypertension and chronic renal disease. Furthermore, negative ANCA, negative serum autoimmune markers, lack of malignancy, and lack of either arthralgia or myalgia ruled out the likelihood of systemic vasculitis (Hernandez-Rodriguez et al. 2008). Because panniculitis and/or vasculitis were not present at other sites or organs at the time of diagnosis or during follow-up, idiopathic isolated BPWV was considered the most likely diagnosis. Bacterial pneumonia might be another possible etiology. Three cases of skin leukocytoclastic vasculitis complicating Klebsiella pneumonia bacteremia have been reported in the English literature (Huang et al. 2013; Lloret et al. 2002; Lum et al. 2000). However, all three cases presented with cutaneous rash or pustulosis instead of a single breast nodule.

To date, seventeen cases of isolated BPWV have been reported without evidence of systemic vasculitis (Hernandez-Rodriguez et al. 2008; Kafantari et al. 2008). Only one of the 17 cases was male. Our case is, thus, the second case of isolated panniculitis with vasculitis of the male breast. Fourteen of 18 cases (77.8%, including our case) were proven by excisional biopsy, mastectomy, or incisional biopsy. The other four cases were proven by core-needle biopsy. Sono-guided core-needle biopsy with an 18-gauge needle could not provide the diagnosis of breast vasculitis in our case. This suggests that excisional biopsy or a larger gauge needle during core-needle biopsy should be used to detect the component of breast vasculitis.

Patients with isolated BPWV have an excellent prognosis with resection alone without systemic corticosteroid or immunosuppressive therapy. They have been reported to have a lower relapse rate (as in our case) compared with breast vasculitis with systemic involvement (Hernandez-Rodriguez et al. 2008).

## Conclusions

Isolated panniculitis with vasculitis of the male breast resembled a malignant lesion on ultrasound due to its heterogeneous hypoechogenicity, partially indistinct margin, echogenic interface and increased internal vascularity. To make an accurate preoperative diagnosis of a male breast mass, a physician must analyze various imaging findings. Sono-guided core-needle biopsy with an 18-gauge needle was insufficient to make the diagnosis of breast vasculitis. Excisional biopsy and clinical data provided the correct diagnosis and determined the appropriate treatment strategy.

### Consent

Written informed consent was obtained from the patient for the publication of this report and any accompanying images.
